# Transposon mediated co-integration and co-expression of transgenes in CHO-DG44 cells

**DOI:** 10.1186/1753-6561-5-S8-P32

**Published:** 2011-11-22

**Authors:** Sowmya Balasubramanian, Mattia Matasci, Lucia Baldi, David L Hacker, Florian M Wurm

**Affiliations:** 1 Laboratory for Cellular Biotechnology (LBTC), Faculty of Life Sciences, École Polytechnique Fédérale de Lausanne CH-1015 Lausanne, Switzerland

## Background

Transposon systems mediate stable integration of exogenous DNA elements into a host cell genome, and have been successfully used in mammalian cells for the generation of stable cell lines. The *piggyBac* (PB) transposon system has been shown to have several advantages over the other transposon system available [1-3]. It has also been shown to generate stable cell lines at significantly higher frequency than the conventional transfections [3]. Here, we investigated the efficiency of the *piggyBac* (PB) transposon to facilitate the co-expression of multiple artificial transposons, each bearing a single transgene and the puromycin resistance gene for selection. Green fluorescent protein (eGFP), red fluorescent protein (mKate), and a human IgG1 antibody were used as model proteins [4]. The effect of the stringency of selection on pool productivity was determined with increasing concentrations of puromycin. The duration of selection necessary for the generation of recombinant cell pools was also tested by selecting for a period of either 5 or 10 days.

## Materials and methods

### CHO-DG44 transfection

Cells were transfected using linear 25 kDa polyethylenimine (PEI) (Polysciences, Eppenheim, Germany). All the transfections are done in a final volume of 10 mL. Transfected cultures were incubated at 37°C in 5% CO_2_ and 85% humidity with agitation at 180 rpm. The ratio of plasmid coding for Gene of Interest (GOI) to the plasmid coding for the transposase was kept constant at 9:1.

### Generation of pools and clones

For the generation of stable pools, two days post transfection the cells were seeded at a density of 5 x 10^5^ cells/mL in ProCHO5 and puromycin. The cells were placed under selection pressure for 5 or 10 days. In case of a 10 day selection period, the puromycin concentration in the cultures was replenished on day 7 post transfection by seeding at a density of 5 x10^5^ cells/mL in fresh ProCHO5 with puromycin. For productivity analysis the cells were seeded at a density of 3 x10^5^ cells/mL and analyzed at day four.

Stable clones were generated by limiting dilution of the pools. The productivity of the clones was analyzed after five days of culture.

### Analyses

A Guava EasyCyte microcapillary flow cytometer (Millipore) with excitation and emission wavelengths of 488 and 532 nm, respectively, was used to measure EGFP-specific fluorescence. The IgG concentration in the culture medium was determined by sandwich ELISA as previously described [4].

## Results

### PB Transposition significantly enhances co-expression of multiple genes from stable pools compared to standard transfection

**Figure 1:**
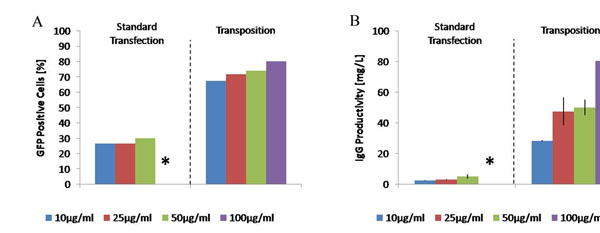
Comparison of the A) %GFP positive cells and B) IgG1 productivity between pools generated using standard transfection (left) and PB transposition (right)

Cells were co-transfected with three artificial transposons namely, eGFP and the heavy and light chains of a human IgG1 antibody along with the plasmid coding for the transposase. Cell populations recovered after selection showed 30 % of GFP positive cells for standard transfections, whereas for pools generated by PB transposition, the percentage of cells producing GFP was up to 80%, which represent a 2.5 fold improvement (Fig. 1A). IgG1 productivity up to 120 mg/L was achieved using transposition, whereas in standard transfections the highest titers obtained were only 5 mg/L, corresponding to a 24 fold improvement (Fig. 1B).

No significant differences were observed in the percentage of GFP positive cells and IgG productivity in cell pools generated by transposition with 5 or 10 days of selection. However, increased IgG productivity and % GFP-positive cells were observed with 10 days of selection in case of standard transfection (data not shown). This shows that a longer duration of selection pressure is necessary to generate pools by standard transfections compared to transposition. Furthermore, increased antibody productivity was observed with increasing puromycin concentration (Fig. 1).

### PB transposition strongly improved clonal productivity

Four different plasmids coding for (1) EGFP, (2) mKATE, (3) the light and (4) heavy chain genes of a human IgG1 antibody. Clones for both the standard and the transposition-based transfections were generated by limiting dilution of the cell pools after selection for 10 days. The IgG productivities of 65 clones of each standard transfection and transposition were analyzed. About 96% of the clones generated by the standard transfection were found to be low-producing. However, more than 40% of clones generated by transposition produced more than 20 mg/L and about 12% of the clones were high-producers (Table 1).

**Table 1:** Distribution of clones based on their IgG productivity

Productivity Range	Standard Transfection	Transposition
**<20 mg/L**	96%	57%
**20-60 mg/L**	4%	30%
**>60 mg/L**	0%	13%

## Conclusions

Based on the above results we conclude that the *piggyBac* transposon system provides an efficient method for the co-integration of multiple genes. The use of the PB transposon system for the co-integration of multiple genes generates a higher frequency of high-producing clones than standard transfection. The GFP-expressing cell population was larger and the volumetric antibody productivity of the pools were higher with higher stringency of selection. A selection period with puromycin of only 5 days was sufficient for the generation of pools from transposon mediated transfection.
